# Anti-proliferative and apoptotic effect of cannabinoids on human pancreatic ductal adenocarcinoma xenograft in BALB/c nude mice model

**DOI:** 10.1038/s41598-024-55307-y

**Published:** 2024-03-18

**Authors:** Trung Quang Le, Nuntana Meesiripan, Suleeporn Sanggrajang, Nuntakan Suwanpidokkul, Piyaporn Prayakprom, Chatchada Bodhibukkana, Vipada Khaowroongrueng, Kankanit Suriyachan, Somchai Thanasitthichai, Attasit Srisubat, Pattamaporn Surawongsin, Anudep Rungsipipat, Siriwan Sakarin, Kasem Rattanapinyopituk

**Affiliations:** 1 Department of Veterinary Pathology, Center of Excellent for Companion Animal Cancer–(CECAC), Chulalongkorn University, Bangkok, 10330 Thailand; 2 The International Graduate Program of Veterinary Science and Technology (VST), Faculty of Veterinary Science, Chulalongkorn University, Bangkok, 10330 Thailand; 3 Faculty of Veterinary Medicine, College of Agriculture, Can Tho University, Can Tho, 94000 Vietnam; 4 Division of Research and Academic Support, National Cancer Institute, Bangkok, 10400 Thailand; 5 The Government Pharmaceutical Organization, Bangkok, 10400 Thailand; 6 Institute of Medical Research and Technology Assessment, Ministry of Public Health, Nonthaburi, 11000 Thailand; 7 Division of Medical Technical and Academic Affairs, Ministry of Public Health, Nonthaburi, 11000 Thailand; 8 Research and Technology Assessment Department, Ophthalmology Department, Lerdsin Hospital, Bangkok, 10500 Thailand

**Keywords:** Anti-proliferation, Apoptosis, Cannabis, Nude mouse xenograft model, Pancreatic adenocarcinoma, Cancer, Drug discovery, Oncology

## Abstract

Human pancreatic ductal adenocarcinoma (PDAC) is a highly malignant and lethal tumor of the exocrine pancreas. Cannabinoids extracted from the hemp plant *Cannabis sativa* have been suggested as a potential therapeutic agent in several human tumors. However, the anti–tumor effect of cannabinoids on human PDAC is not entirely clarified. In this study, the anti–proliferative and apoptotic effect of cannabinoid solution (THC:CBD at 1:6) at a dose of 1, 5, and 10 mg/kg body weight compared to the negative control (sesame oil) and positive control (5-fluorouracil) was investigated in human PDAC xenograft nude mice model. The findings showed that cannabinoids significantly decreased the mitotic cells and mitotic/apoptotic ratio, meanwhile dramatically increased the apoptotic cells. Parallelly, cannabinoids significantly downregulated Ki-67 and PCNA expression levels. Interestingly, cannabinoids upregulated BAX, BAX/BCL-2 ratio, and Caspase-3, meanwhile, downregulated BCL-2 expression level and could not change Caspase-8 expression level. These findings suggest that cannabinoid solution (THC:CBD at 1:6) could inhibit proliferation and induce apoptosis in human PDAC xenograft models. Cannabinoids, including THC:CBD, should be further studied for use as the potent PDCA therapeutic agent in humans.

## Introduction

Pancreatic cancer has been estimated that it will be the second cause of cancer-associated mortality in the next decade^1,2^. Pancreatic ductal adenocarcinoma (PDAC) is a highly malignant tumor and the most common neoplasm in the exocrine pancreas, approximately appearing in 90% of all pancreatic malignancies^1,3,4^. Even though several therapeutic drugs were prior developed, suitable therapeutic drugs for PDAC are still a major challenge for scientists worldwide. One of the most concerns in the treatment of human PDAC is a poor response at the late stage, resistance exhibited, and the side effects of the therapeutic agents^1,4^. Therefore, continuously investigating novel, safe, and effective treatments for human pancreatic cancer is still necessarily needed.

Herbal medicinal plants and their derivatives have been discovered and used as potential sources for the treatment of human cancers for decades. Of these, cannabinoids extracted from the hemp plant *Cannabis sativa* have been remarkably noted as a potential therapy for the treatment of several human tumors^5–10^. The anti–tumor effect of cannabinoids was reported in four main ways, including (1) inhibition of tumor cell proliferation, (2) induction of apoptosis, (3) inhibition of angiogenesis, invasion and metastasis, and (4) induction of anti-tumor immunity^11,12^. At present, even though over 60 cannabinoid compounds were recorded, Delta-9-tetrahydrocannabinol (THC) and cannabidiol (CBD) have been widely acknowledged as one of the most active compounds^11,13^. Notably, the previous studies indicated that the combination between THC and CBD exerted an increase in anti-tumor effects compared with the single use of individual components^14–16^. However, the anti–tumor effect of cannabinoids (THC combined with CBD) on pancreatic cancer animal models is understudied. The goal of this study therefore to evaluate the anti-proliferative effect and the apoptosis induction in pancreatic cancer xenograft mice treated with cannabinoids.

## Results

### Gross and histopathological findings

Grossly, the tumors were presented subcutaneously at the transplanted areas of all xenograft nude mice in each group. In the xenograft tumors, ulcerated and necrosis were found in several nude mice of each experimental group (Supplementary data Fig. 1). In other internal organs, there was no significant change in gross examination except mild to moderate pulmonary congestion. The metastasis of tumor cells in both intrathoracic and intraabdominal organs was not macroscopically observed. Besides, we also did not observe a significant change in both hematology and blood chemistry profiles among different experimental groups.

The average tumor volume (mm^3^) was calculated on day 0 (initial day) and day 30 (final day) of the experiment. There was no significant difference (*p* > 0.05) between the average volume of the xenograft tumors over the course of treatment. Furthermore, there was no statistical difference (*p* > 0.05) in the percentage of tumor volume change among groups (Supplementary data Fig. 2).

To evaluate the histopathological morphology of the transplanted tumors on the xenograft nude mice, the H&E–stained section of an individual xenograft tumor was examined and evaluated under a light microscope (40×). The xenograft tumors were arranged into the glandular structure with fibrovascular stroma surrounding. Neoplastic cells were round to oval, lobulated with the intralesional necrotic area, and surrounded by clearly defined borders. The necrotic areas were frequently represented on the edge of xenograft tumors in the treatment and PC groups, while occasionally found in the center of the xenograft tumor in the NC group. The transplanted tumors displayed similar characteristics to the original neoplasm which was pancreatic ductal adenocarcinoma. In subtype classification, all the examined tumors mirrored the characteristics of adenosquamous carcinoma according to the World Health Organization (WHO) classification (Supplementary data Fig. 3). The metastasis of tumor cells in both intrathoracic and intraabdominal organs was not microscopically observed. Besides, there was no significant change in the collected organs, except mild pulmonary congestion (Supplementary data Fig. 4). Moreover, this study performed immunohistochemistry (IHC) to detect the proliferation of blood vessels in the xenograft tumors via a specific antibody (CD31/PECAM-1). The immunoexpression of the CD31/PECAM-1 antibody showed no significant proliferation (*p* > 0.05) of the blood vessels in the xenograft tumors among groups (Supplementary data Fig. 5).

### Mitotic and apoptotic index

**Figure 1:**
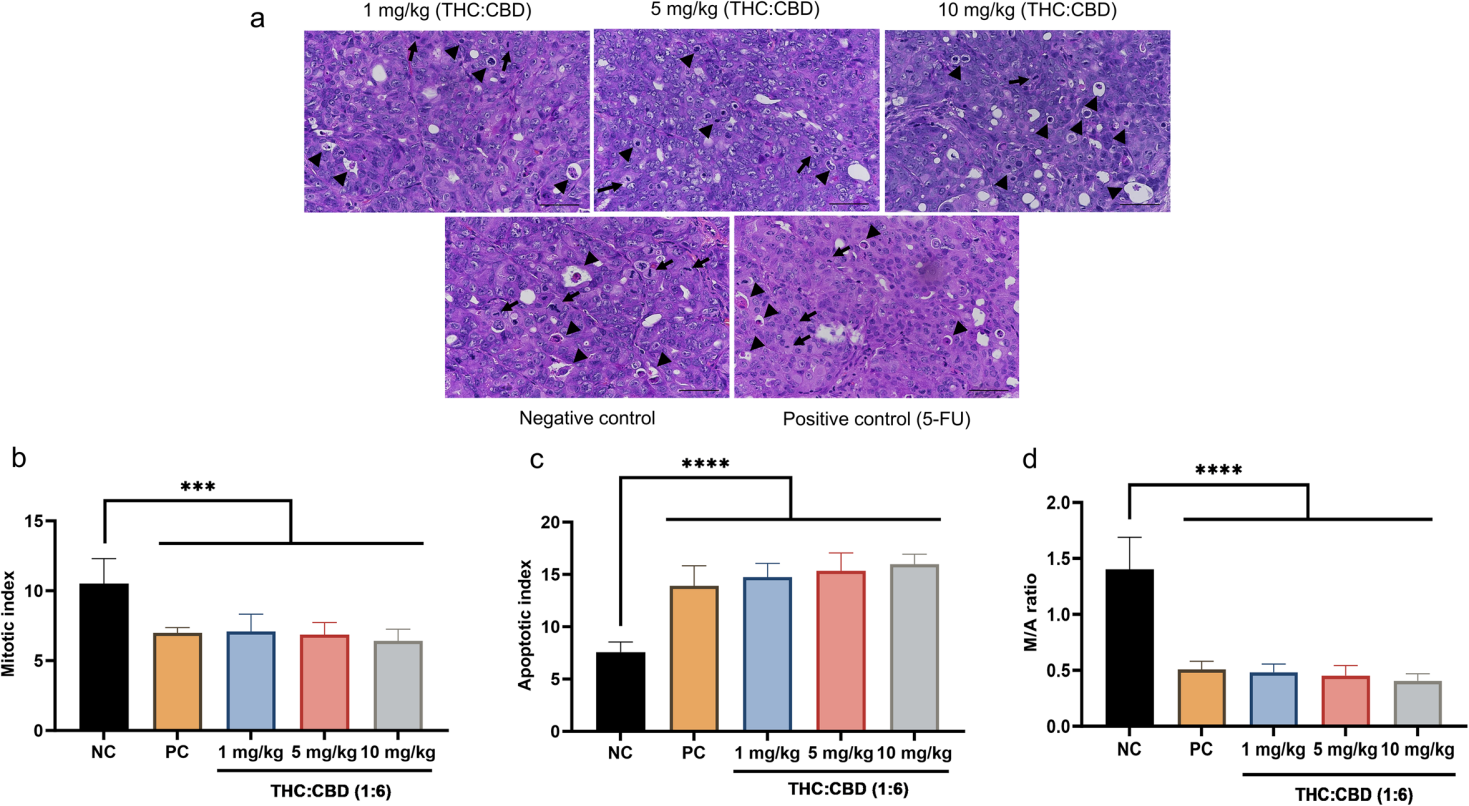
Effect of cannabinoids on the mitotic and apoptotic index. (**a**) The xenograft tumors performed scattered mitotic cells (arrow) and apoptotic cells (arrowhead) (H&E stained, 40×, scale bar = 50 µm). (**b**–**d**) The mitotic (**b**), apoptotic cell count (cells/HPF) (**c**), and M/A ratio (**d**) among the NC group, PC group, and cannabinoids–treated groups at a dose of 1, 5, and 10 mg/kg BW. The values were presented as mean ± SD. ***p < 0.01; ****p < 0.001 (one–way ANOVA and post hoc test).

In the area of the predominated number of apoptotic cells, the number of mitotic cells was rare (Fig. 1a). For mitotic cell count, the number of mitotic cells (cells/high–power field (HPF)) was highest in the negative control (NC) group compared with those in cannabinoid–treated groups and the positive control (PC) group. The difference in the mitotic cell count between the NC group and experimental groups was statistically significant (*p* < 0.01) (Fig. 1b). In contrast, the apoptotic cell count (cells/HPF) was significantly increased (*p* < 0.001) in cannabinoid–treated groups, and the PC group relative to the NC group (Fig. 1c). When comparing the mitotic/apoptotic (M/A) ratio, there was dramatically decreased (*p* < 0.001) in the PC group and cannabinoid–treated groups compared to the NC group (Fig. 1d).

### Apoptotic-related gene expression

**Figure 2:**
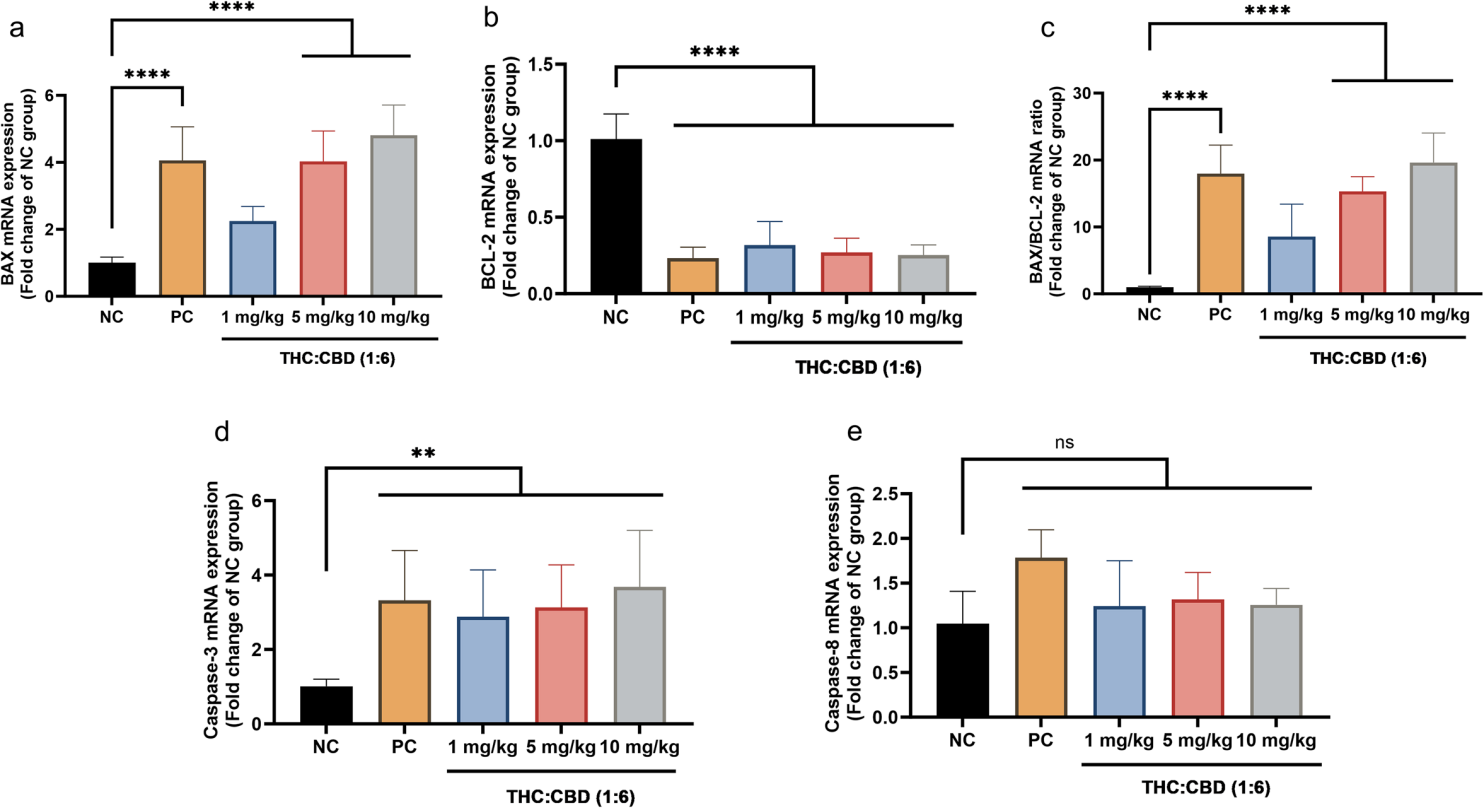
The mRNA expression levels of *BAX* gene (**a**), *BCL-2* gene (**b**), *BAX*/*BCL-2* ratio (**c**), *Caspase-3* gene (**d**), and *Caspase-8* gene (**e**) among the NC group, PC group, and cannabinoids-treated groups at a dose of 1, 5, and 10 mg/kg BW. The values were presented as mean ± SD. **p < 0.05; ****p < 0.001; ^ns^*p* > 0.05 (Kruskal–Wallis test).

To investigate the effect of cannabinoids on the human PDAC xenograft tumors through the mechanism of tumor cell death, the mRNA expression level of apoptotic–related genes including *BAX*, *BCL-2*, *Caspase-3*, and *Caspase-8* was performed. As demonstrated in the results, the relative expression level of the pro–apoptotic *BAX* gene was significantly different (*p* < 0.001) in the PC and cannabinoid-treated groups at a dose of 5 and 10 mg/kg body weight (BW), whereas there was no statistical difference (*p* > 0.05) in cannabinoid–treated group at a dose of 1 mg/kg BW relative to the NC group (Fig. 2a). The relative expression level of the anti-apoptotic *BCL-2* gene was dramatically lower (*p* < 0.001) in the PC and cannabinoid–treated groups compared to the NC group (Fig. 2b). The *BAX*/*BCL-2* relative expression ratio significantly rises (*p* < 0.001) in the PC and cannabinoid-treated groups at a dose of 5 and 10 mg/kg BW relative to the NC group (Fig. 2c). However, the relative expression level of the *BAX*/*BCL-2* ratio in cannabinoid-treated group at a dose of 1 mg/kg BW was not significantly different (*p* > 0.05) in comparison to the NC group (Fig. 2c). The relative expression level of the pro-apoptotic *Caspase-3* gene dramatically increased (*p* < 0.05) in the PC and cannabinoid–treated groups relative to the NC group (Fig. 2d). Moreover, the relative expression level of the pro–apoptotic *Caspase-8* gene was not significantly different (*p* > 0.05) among PC, cannabinoid–treated groups, and the NC group (Fig. 2e).

### Immunohistochemical examination

**Figure 3:**
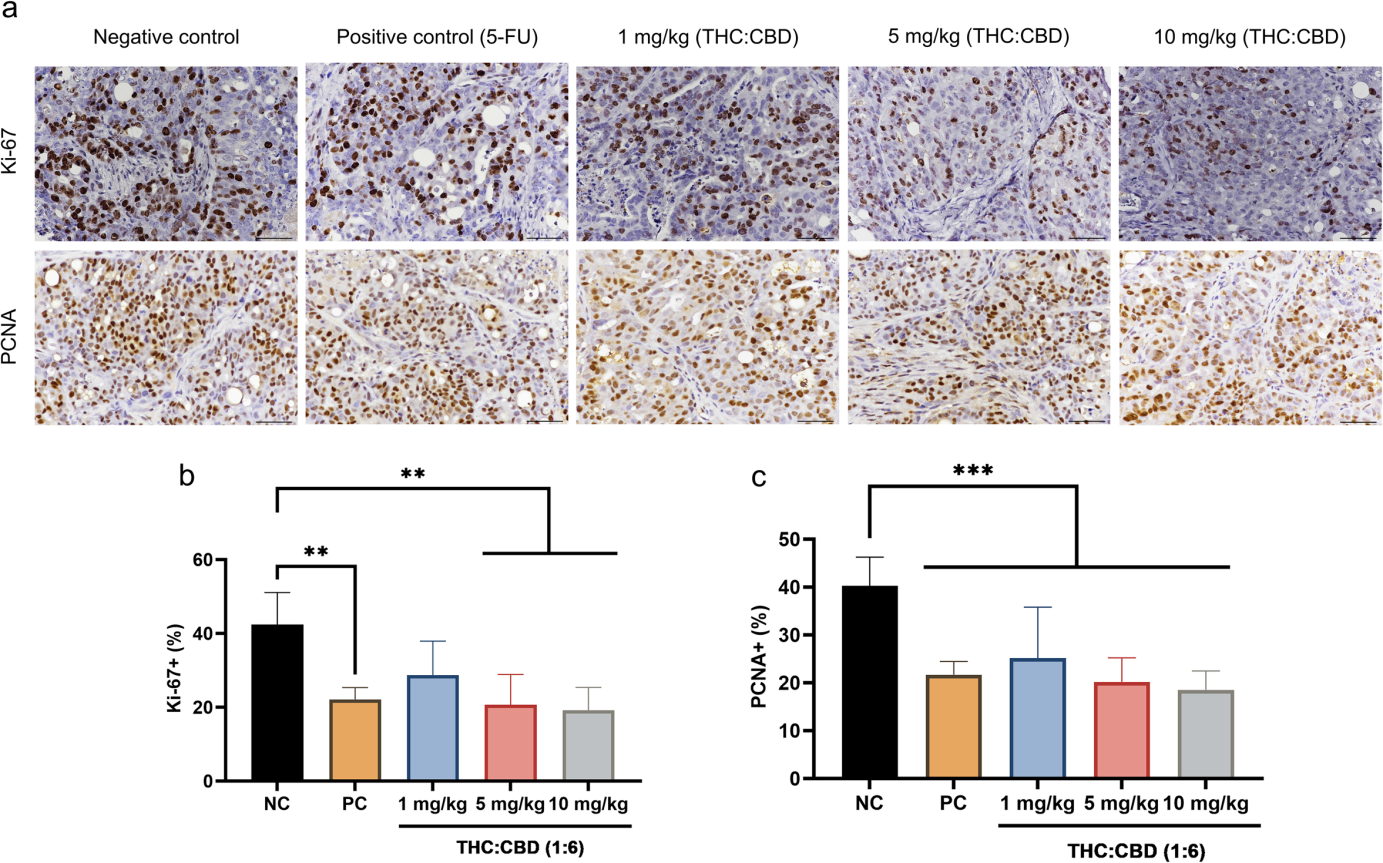

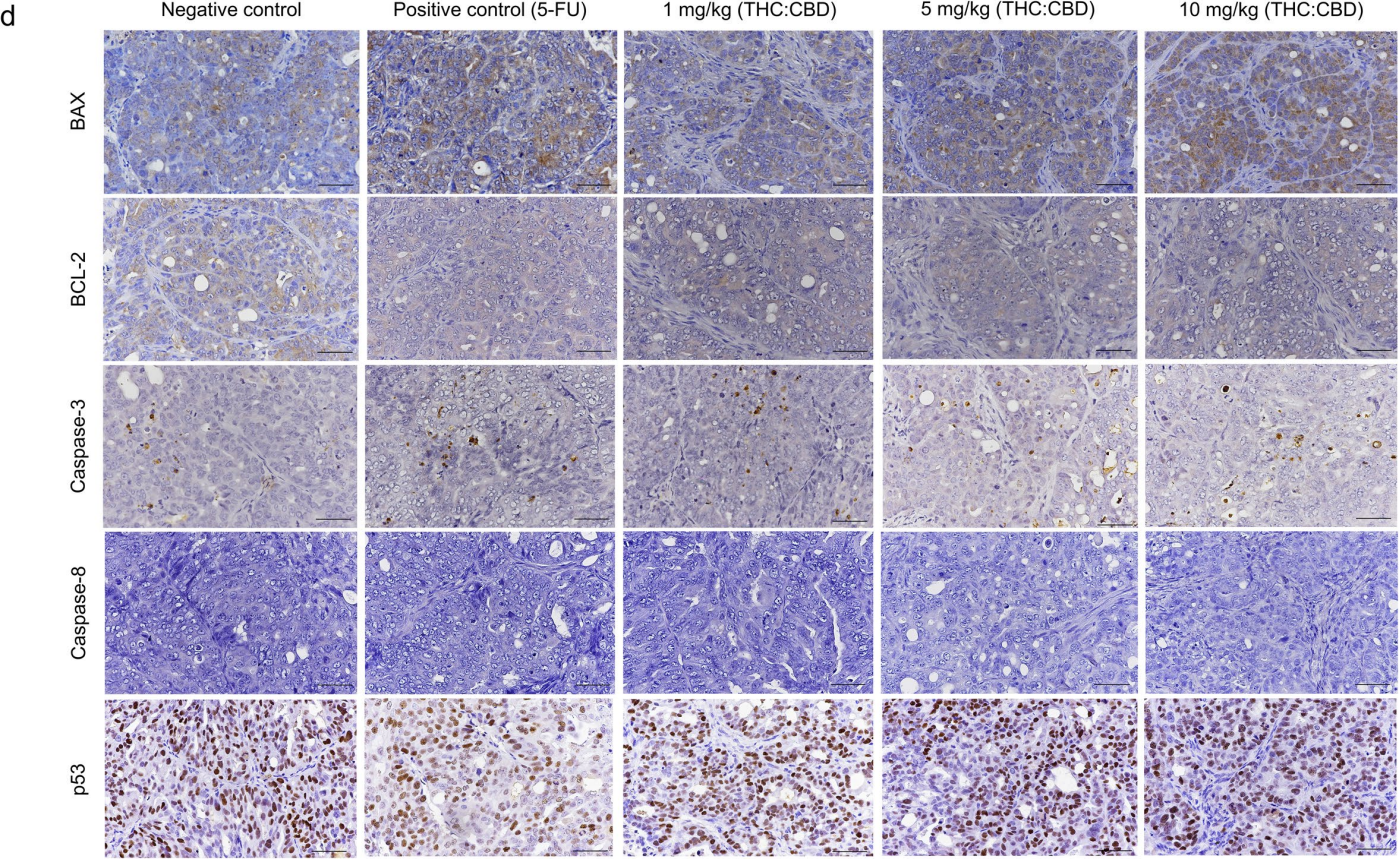

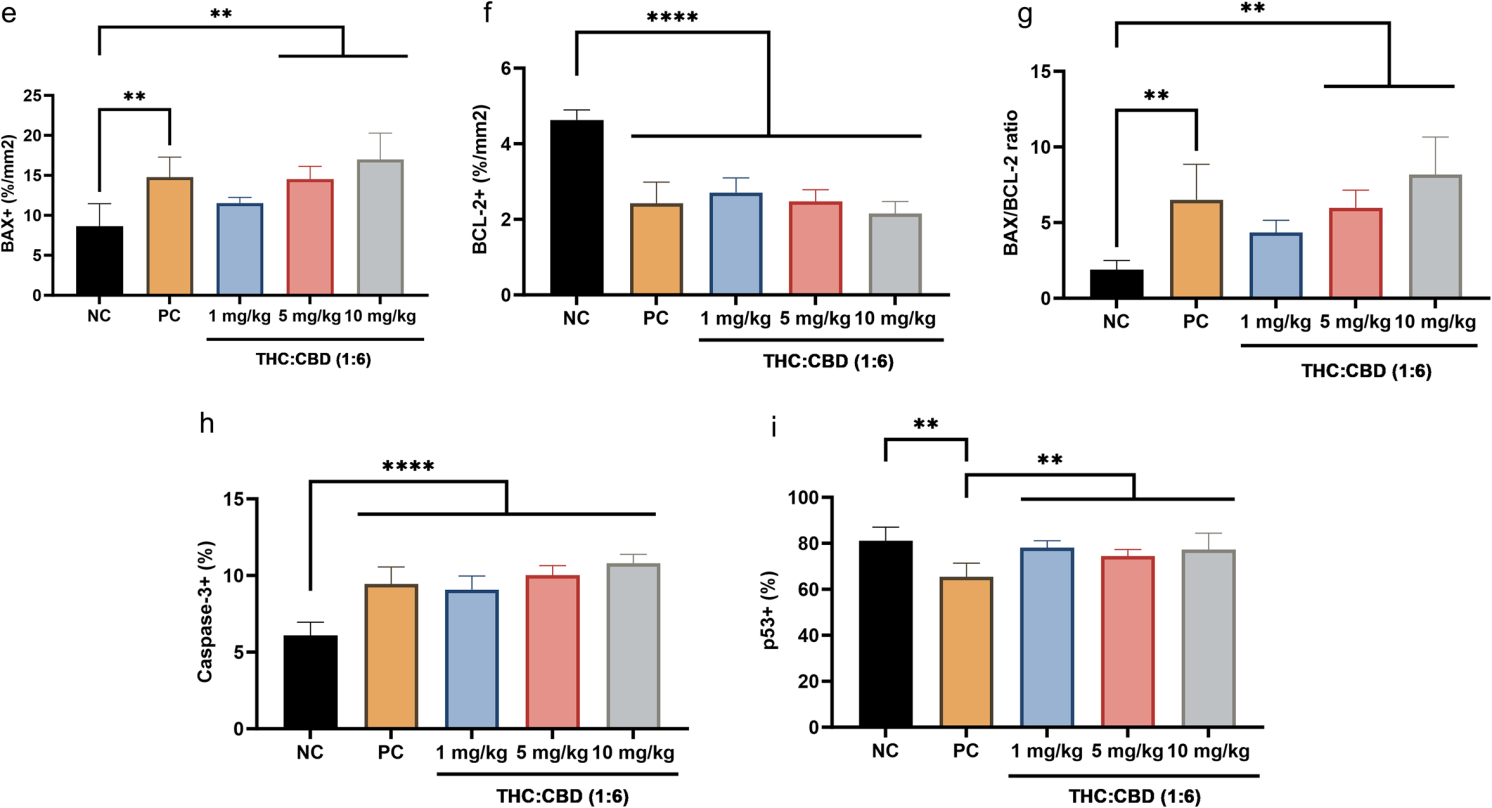
The immunoexpression of anti-proliferative markers (Ki-67 and PCNA) (**a**), and anti–apoptotic markers (BAX, BCL-2, Caspase-3, Caspase-8, and p53) (**d**) among the NC group, PC group, and cannabinoids–treated groups at a dose of 1, 5, and 10 mg/kg BW. The intranuclear labeling of Ki–67, PCNA, Caspase-3, and p53; intracytoplasmic labeling of BAX, BCL-2 in the xenograft tumors were displayed as brown color (DAB labeled, Mayer’s hematoxylin counterstained, 40×, scale bar = 50 µm). The expression levels of protein Ki-67 (**b**), PCNA (**c**), BAX (**e**), BCL–2 (**f**), BAX/BCL-2 ratio (**g**), Caspase-3 (**h**), and p53 (**i**) among the NC group, PC group, and cannabinoids–treated groups at a dose of 1, 5, and 10 mg/kg BW. The values were presented as mean ± SD. **p < 0.05; ***p < 0.01; ****p < 0.001 (Kruskal–Wallis test).

Correlation with the aim to investigate the effect of cannabinoids on the human PDAC xenograft tumors via the mechanism of tumor cell death, IHC was carried out to detect the expression of proliferative markers (Ki-67 and PCNA), and apoptotic–related markers (BAX, BCL-2, Caspase-3, Caspase-8, and p53) (Fig. 3a,d). As presented in the results, the percentage of Ki-67-positive labeling cells was significantly reduced (*p* < 0.05) in cannabinoid–treated group at a dose of 5, 10 mg/kg BW, and the PC group, meanwhile there was not statistically different (*p* > 0.05) in cannabinoid–treated group at a dose of 1 mg/kg BW relative to the NC group (Fig. 3b). However, the percentage of PCNA–positive labeling cells was dramatically different (*p* < 0.01) among cannabinoid–treated groups and the PC group when compared with the NC group (Fig. 3c). On the other hand, consistent with the gene expression results, cannabinoids and the PC groups significantly increased (*p* < 0.05) the expression of BAX protein, BAX/BCL-2 ratio, meanwhile statistically decreased (*p* < 0.001) the expression of BCL–2 protein and Caspase-3 protein (Fig. 3e–h). However, the expression of BAX protein and BAX/BCL-2 ratio was not different (*p* > 0.05) between cannabinoid–treated group at a dose of 1 mg/kg BW and the NC group (Fig. 3e,g). Furthermore, there was no positive labeling of Caspase-8 protein in all groups (Fig. 3d). For p53 protein expression, the percentage of positive labeling cells was significantly reduced (*p* < 0.01) in the PC group, meanwhile there was not statistically different (*p* > 0.05) in cannabinoid–treated groups compared to the NC group (Fig. 3i).

### Western blot analysis

**Figure 4:**
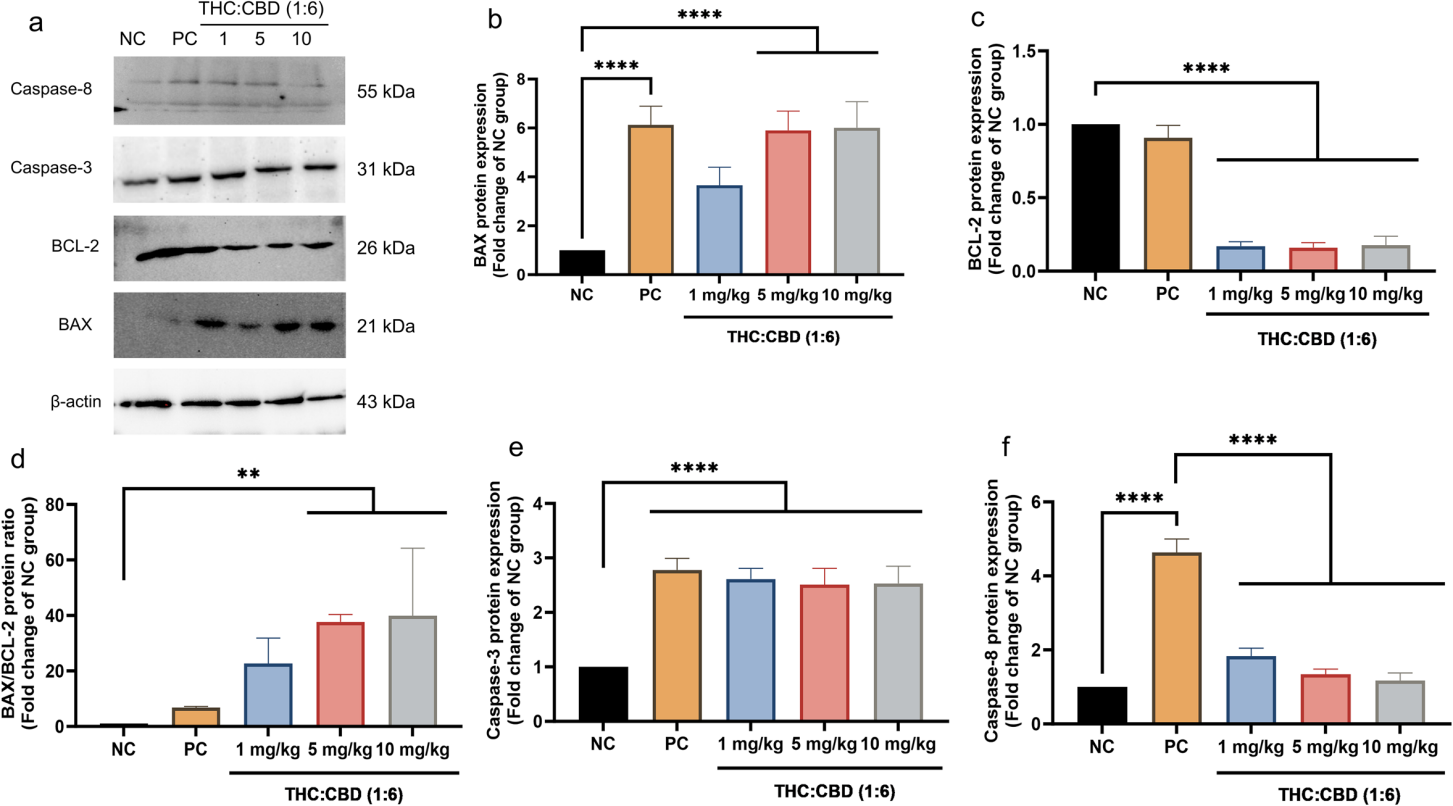
The representative WB results (**a**) and relative expression levels of protein BAX (**b**), BCL–2 (**c**), BAX/BCL–2 ratio (**d**), Caspase-3 (**e**), and Caspase-8 (**f**) among the NC group, PC group, and cannabinoids–treated groups at a dose of 1, 5, and 10 mg/kg BW. The values were presented as mean ± SD. **p < 0.05; ****p < 0.001 (Kruskal–Wallis test).

The Western blot (WB) was conducted to confirm the gene and IHC expressions. These results showed that the relative protein expression levels of BAX, BAX/BCL-2 ratio, and Caspase-3 increased significantly (*p* < 0.05 or *p* < 0.001) in cannabinoid–treated groups and the PC group relative to the NC group (Fig. 4b, d, and e). Contradictorily, the relative protein expression levels of BAX in cannabinoid-treated group at a dose of 1 mg/kg BW were not significantly different (*p* > 0.05) compared to the NC group (Fig. 4b). Meanwhile, the relative protein expression levels of BCL–2 were dramatically decreased (*p* < 0.001) in cannabinoid-treated groups compared to the NC group (Fig. 4c). Besides, the Caspase-8 protein expression levels were dramatically increased (*p* < 0.001) in the PC group when compared with the NC group, meanwhile, there was not statistically significant (*p* > 0.05) in cannabinoid-treated groups and the NC group (Fig. 4f).

## Discussion

In recent years, THC and CBD have been popularly accepted as the most active ingredient of cannabinoids derived from *Cannabis* plants^11,13^. Several studies on various human cancers showed a higher effect when THC was combined with CBD^14–17^. In the current study, the combination of THC:CBD with a ratio of 1:6 was investigated the anti-tumor effects within a human PDAC cell line (Capan-2) derived xenograft nude mice model. The findings of this study illustrated that cannabinoids (THC:CBD) at a ratio of 1:6 could inhibit proliferation and induce apoptosis in human PDAC xenograft nude mice models.

Herein, the examination of xenograft tumors displayed that the increase of THC:CBD dose did not affect the percentage of tumor volume change among groups. The findings of the current study imply that cannabinoids could induce apoptosis in the PDAC cells, however, there was no effect on normal pancreatic cells. Our results were similar to the prior study^18^, activating cannabinoid receptors could induce apoptosis in pancreatic cancer cells but did not affect normal pancreatic cells. Moreover, the metastatic spread of the human PDAC cell line transplanted into the xenograft mice and the histopathological change in the internal organs were examined by evaluation in the H&E-stained images of both intrathoracic organs (lung, heart) and intraabdominal organs (liver, pancreas, spleen, kidneys). As shown in the results, there was no significant change in the selected organs, except mild pulmonary congestion. Evidence of inflammation and necrosis in the representative organs was not discovered. These findings suggested that there was no metastatic spread of the human PDAC cell line to the examined organs.

The anti–tumor effect of cannabinoids has been partly reflected via inhibiting tumor cell proliferation. It is shown that cannabinoids modulated cell cycle checkpoints in the tumor cells through the cannabinoid receptors, consequently blocking tumor cell proliferation^11,12^. Regarding this study, the histopathological findings showed that the increase of THC:CBD dose led to the rise of apoptotic index while the reduction of mitotic index among groups. The M/A analysis indicated that higher doses of cannabinoids fell down the M/A ratio among groups. Consistent with the mitotic index, we observed that the percentage of Ki-67 and PCNA-positive cells dramatically dropped in cannabinoid-treated groups. For decades, nuclear antigen Ki-67 and PCNA were recruited as the most popular proliferation markers for numerous human cancer studies worldwide^19–22^. It is noteworthy that the combination of Ki-67 and PCNA improved the sensitivity and specificity in the diagnosis of proliferated cells^23,24^. In fact, Ki-67 and PCNA were widely used in clinical diagnosis and laboratory animal studies of human cancers, especially within the xenograft mice model^21–24^. Interestingly, the current study showed that cannabinoids at the lowest dose (1 mg/kg BW) could not affect the expression of Ki–67 protein in human PDAC. Indeed, a prior study demonstrated that the combination of THC and CBD reduced the expression of Ki-67 in human glioblastoma cells^25^. It is believed that THC could be associated with the activation of cannabinoid receptors (GPR55), meanwhile, antagonistic results were observed in CDB, resulting in a decrease in Ki-67 expression^25^. Based on our results, the interaction between cannabinoids and the expression of Ki-67 should be addressed in further studies. Taken together, the current study implies that THC:CBD (1:6) could inhibit PDAC cell proliferation in xenograft nude mice models.

On the other hand, cannabinoids have been related to the part of the apoptosis induction within various tumors, especially PDAC^6,11,26^. Apoptosis is a complex physiological process that occurs as the homeostatic mechanism to ensure the balance of cell populations in the tissue of eukaryotes. There are two principal pathways to activate apoptosis that stimulate the intrinsic or extrinsic pathway. In both pathways, it is noted that several involved proteins including BAX, BCL-2, Caspase-3, Caspase-8, and p53^27–29^. The findings of this study indicated that cannabinoids induced apoptosis by upregulation of BAX and Caspase-3, downregulation of BCL-2, and may not be involved with the activation of Caspase-8 and p53 in human PDAC xenograft model. These findings were similar to the results of the previous studies that apoptosis induced by cannabinoids was strictly related to the intrinsic pathway^6^.

The upregulation of pro-apoptotic protein BAX is associated with the permeabilization of the mitochondrial outer membrane, permitting the release of cytochrome C, thus promoting mitochondrial apoptotic via an intrinsic pathway^29–31^. In this study, both mRNA and protein expression of BAX markedly increased in cannabinoid–treated groups at a dose of 5 mg/kg BW and 10 mg/kg BW while low expressed in the NC group. Our results were in agreement with the earlier study that cannabinoids were associated with the upregulation of BAX levels^32,33^. Besides, the current study showed that the lowest dose of cannabinoids (1 mg/kg BW) could not affect the expression of BAX. Altogether, it implies that apoptosis of human PDAC xenograft tumor was involved with the upregulation of BAX protein compared to the NC group.

The anti-apoptotic protein BCL-2 is believed to inhibit the activity of protein BAX, thus preventing downstream activation of apoptosis via an intrinsic pathway^29–31^. According to this study, the mRNA and protein expression of BCL-2 significantly dropped in the treatment groups compared to the NC group. Clinically, a previous study conducted on PDAC demonstrated that the upregulation of BCL–2 level was inversely correlated with the overall survival time of the patients^34^. It is implied that silencing of BCL-2 may stimulate apoptosis in cancer cells^34,35^. Likewise, recent studies reported that cannabinoids downregulated BCL–2 levels in mouse pancreatic β cell lines and human gastric cancer cell lines^26,33^. The obtained results displayed that the downregulation of BCL-2 levels is associated with the activation of apoptosis in human PDAC xenograft tumors.

Remarkably, BAX/BCL–2 ratio has been widely accepted as a protein expression pattern to estimate the eventual outcome of apoptosis expression in cancer patients^36–38^. An imbalance of protein BAX and BCL-2 expression may associate with the activation of the apoptosis process in cancer cells^29,30,33^. However, individual use of BAX and BCL–2 expression patterns could be associated with unclear evidence of apoptosis expression^37^. Therefore, the current study additionally utilized this ratio to assess the apoptosis expression in the human PDAC–derived xenograft nude mice model. As presented in our results, the BAX/BCL-2 ratio was significantly different between the cannabinoid–treated groups and the NC group, except the cannabinoid–treated group at a dose of 1 mg/kg BW. Considering individual expression of BAX and BCL-2 protein showed the upregulation of protein BAX and downregulation of protein BCL-2 in the treated groups compared to the NC group. The consequence was an increased BAX/BCL-2 ratio in cannabinoid–treated groups. The increase in BAX/BCL-2 ratio reflected the critical confirmation of tumor cell death^36–38^. Similarly, a previous study demonstrated that CBD stimulated apoptosis via downregulation of protein BCL-2 and upregulation of protein BAX in gastric cancer cell lines^33^. Thus, this result confirms that cannabinoids could promote apoptosis in the human PDAC xenograft model with the upregulated BAX, downregulated BCL-2, and upregulated BAX/BCL-2 ratio.

Moreover, the activity of protein p53 was additionally investigated in this study. Protein p53 plays a central role in promoting apoptosis via the regulation of protein BAX and BCL-2 within the intrinsic pathway^27,30,39^. However, the present study showed that there were no significant differences between cannabinoid–treated groups and the NC group. In fact, the mutant p53 gene was frequently reported in primary human PDAC and wild-type for the Capan-2 cell line^40,41^. It is believed that the Capan-2 cell line possessing wild-type p53 could affect the interaction of cannabinoids. Further studies should be noted the role of protein p53 in the activation of apoptosis program on the human PDAC (Capan-2) cell line.

Pro–apoptotic protein Caspase-3 has been well known as one of the most important proteins in the apoptosis process. Both intrinsic and extrinsic apoptosis pathways have converged to Caspase-3. Activation of the Caspase-3 protein has directly related to the response of apoptosis in cancer cells^27,29,39,42^. This study found that the mRNA and protein expression level of Caspase-3 was significantly increased in the treatment groups with cannabinoids in comparison to the NC group. Consistent with previous studies, cannabinoids upregulated protein Caspase-3, resulting in activated apoptosis^16,32,33,43–45^. For human pancreatic cancer, protein Caspase-3 was activated by cannabinoids in in vitro study, which ultimately resulted in apoptosis^6^. Similarly, the obtained results of this study once again confirm that cannabinoids stimulate apoptosis in the human PDAC xenograft model by upregulation of protein Caspase-3.

Contrarily, the current results presented that cannabinoids could not relate to the mRNA and protein expression of Caspase-8. Meanwhile, the expression of protein Caspase-8 was not detected via IHC in all groups. Caspase-8 – a pro–apoptotic protein triggered by outside stimuli through death receptors in the cell membrane. The Caspase-8 expression has related to the extrinsic apoptotic pathway^27–29^. On the other hand, the earlier investigation showed that cannabinoids were involved with the activation of the intrinsic apoptosis pathway^43^. Likewise, the current finding implies that cannabinoids induced apoptosis in the human PDAC xenograft tumors which may not be associated with the activation of Caspase-8. Taken together with the findings of BAX, BCL–2, and Caspase-3, these data suggest that apoptosis induced via the treatment of cannabinoids in human PDAC xenograft tumors was rather involved with the intrinsic apoptosis pathway.

The present study encountered unavoidable limitations. First, we developed this study from the prior project, all xenograft tumors were inherited from the previous animal experiment. However, the present work was carried out to address the limitations of the prior study and was separated from the initial project. Accordingly, it is believed that effectuating this study is necessarily required. Second, the fluorescence assays to demonstrate the DNA fragmentation in apoptotic cells were not performed in the current study. Several studies mentioned the fluorescence assays to detect fragmented DNA strands such as a terminal deoxynucleotidyl transferase–mediated deoxyuridine triphosphate nick end labeling (TUNEL) assay or Hoechst dye^16,46,47^. These assays could help to increase the reliability of the study by clearly indicating cell death via fluorescence signals. Nevertheless, the immunoexpression of apoptotic–related proteins in both intrinsic and extrinsic pathways (BAX, BCL–2, Caspase-3, and Caspase-8) has been reflected as a powerful tool and has generally been applied to evaluate program cell death^39,48–50^. Besides, the current study recruited WB which is widely mentioned as a gold standard for assessing protein expressions^51,52^. Therefore, the combination of IHC and WB results is probably sufficient to evaluate apoptosis in human PDAC xenograft tumor cells. For further studies, the limitations of this study should be noticed and addressed.

In summary, this study revealed that cannabinoids (THC:CBD) (1:6) could inhibit the proliferation and induce apoptosis in human PDAC xenograft nude mice models. Furthermore, the study additionally illustrates that the anti–apoptotic effect of cannabinoids on the human PDAC xenograft nude mice model could be associated with the intrinsic pathway. With obtained results, the current study contributes new insights to the fundamental knowledge regarding the potential therapeutic role of cannabinoids for human PDAC cell lines derived xenograft nude mouse model. This study may additionally contribute to accommodating more therapeutic targets for human–occurring pancreatic adenocarcinoma in the future.

## Materials and methods

### Preparation of cannabinoid solution

The cannabinoid solution with a ratio of THC:CBD (1:6) was used in this study according to the formula of the Government Pharmaceutical Organization, Thailand. The present study used the ratio of THC:CBD (1:6) followed by the previous publication^53^.

### Animal study

**Figure 5:**
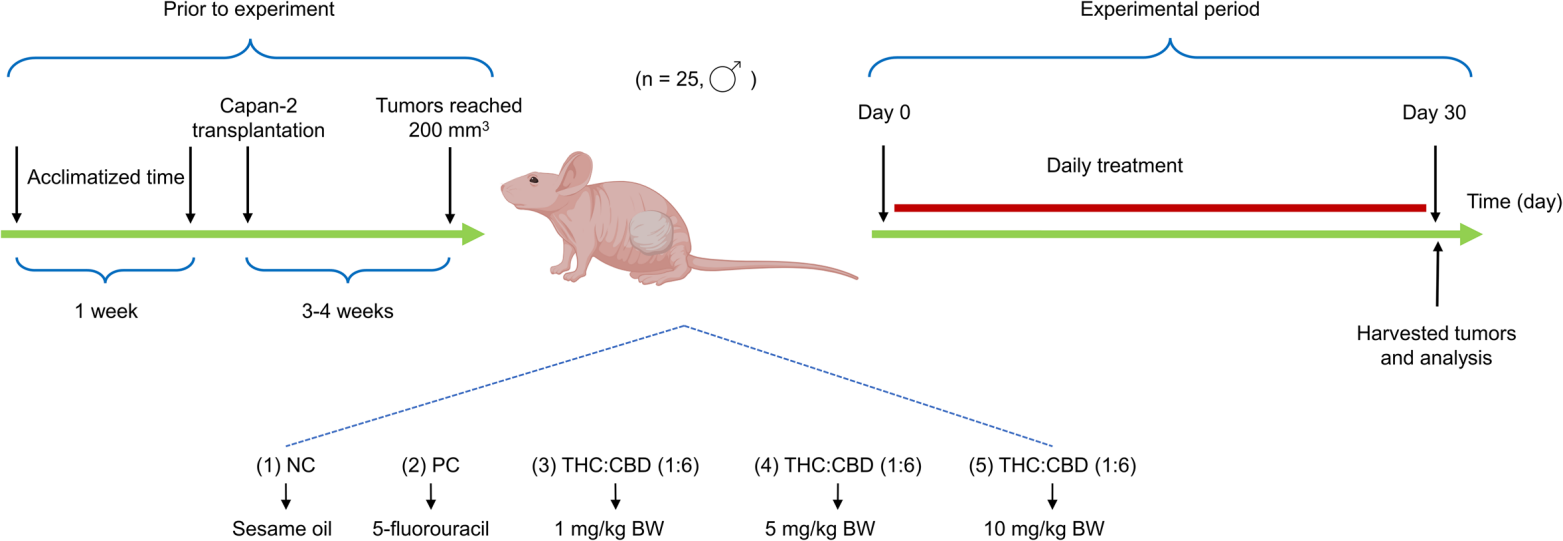
The experimental scheme of this study.

All human PDAC xenograft tumors of this study were recruited from the prior study^53^. The animal experiment was approved by the Institutional Animal Care and Use Committee (IACUC) of The National Cancer Institute, Thailand (Approval No.272_2019RB_IN602) and Lerdsin Hospital, Department of Medical Services (Approval No.ACE–F–v03–02). All animal experiment procedures were completely followed according to the approved guidelines and ARRIVE guidelines. Briefly, 25 male immunodeficient mice (nude mouse, BALB/cAJcl–nu) at 4-weeks-old were acclimatized for one week at a strictly hygienic conventional laboratory system. All mice were subcutaneously injected with 5 × 10^6^ human PDAC (Capan–2, HBT–80) cells at the right flank region under aseptic conditions. The xenograft tumors were measured once every 3 days by a vernier caliper. Tumor volume was calculated according to the described formula: Volume (mm^3^) = (Length x Width^2^)^54^. The treatments were started during the tumor volume developed around 200 mm^3^. Five mice were randomly arranged into a treatment group according to the completely randomized design. Two controls and three experimental treatments were included (1) NC group: mice were gavaged with sesame oil; (2) PC group: mice were intraperitoneally injected with 5–FU at a dose of 20 mg/kg BW, 3 times per week; (3), (4), (5) experimental group 1, 2, 3: mice were gavaged with cannabinoid solution at a dose of 1, 5 and 10 mg/kg BW/day, respectively. The experimental scheme is presented in Fig. 5.

The xenograft tumors and internal organs (lung, heart, liver, pancreas, spleen, and kidney) of experimental nude mice were collected and fixed with 10% neutral buffered formalin for 24 h subsequent to euthanasia, necropsy, and gross examination. In all collected samples, routine tissue processing was performed including paraffin embedding and H&E stain. The morphological evaluation and subtype classification of PDAC were accomplished according to the previous publication of the WHO^55^.

### Mitotic and apoptotic cell count

The number of mitotic and apoptotic cells in individual tumor sections was counted as described in the previous study^56^. For the cell count, a total of ten areas were randomly captured at an HPF (40x, area equals 2.37 mm^2^) per section. Mitotic and apoptotic cell counts were accomplished by manually counting in each group. The average number of mitotic and apoptotic cells per section was followed by the formula of the number of expressed cells per HPF (cells/HPF). The mitotic/apoptotic (M/A) ratio was calculated via dividing the mitotic index by the apoptotic index. A double–blind study was performed to evaluate the results by certified veterinary pathologists.

### Quantitative reverse transcription real–time PCR (qRT–PCR)

The innuPREP RNA Mini Kit 2.0 (Analytik Jena AG, Jena, Germany) was selected to extract the total RNA from fresh xenograft tumors following the manufacturers’ instructions. The aqueous phase of the final mixture was collected and kept at − 80 °C until used for further processing. The synthesis of cDNA was transfected by using the qPCRBIO cDNA Synthesis Kit (PCR Biosystems, London, United Kingdom). The synthesized process followed the manufacturers’ instructions. The concentration of total RNA and synthesized products was checked by the NanoDrop Lite spectrophotometer (Thermo Fisher Scientific Inc., Wilmington, USA).

The expression of apoptotic–related genes was amplified using the gene–specific primer pairs (Table 1) as focusing the previous publications^57–60^. qRT–PCR was performed on a Rotor–Gene Q thermocycler (Qiagen, Hilden, Germany). Briefly, 20 ng of cDNA were well mixed in 10 µL of 2 × qPCRBIO SyGreen Mix (PCR Biosystems, London, United Kingdom), 400 nM of individual primer, and adjusted into a total of 20 µL final volume, according to the manufacturers’ instructions. The thermal cycle of the qRT–PCR reaction consisted of polymerase activation at 95 °C for 2 min, following 40 cycles of denaturation at 95 °C for 5 s, and extension for 30 s at 60 °C. qRT–PCR determined the cycle threshold (Ct) value of individual target genes. An individual sample was carried out in duplicate per each target gene. The relative quantification among target genes was accomplished through the earlier 2^–ΔΔCt^ method^61^. *β–actin* gene was chosen as an internal control following the prior descriptions^62,63^. The expression level of the individual target genes among experimental groups was averaged and displayed as a normalized ratio. The *BAX*/*BCL–2* ratio was calculated by dividing the normalized ratio of *BAX* and *BCL–2* gene expression.

**Table 1:** The sequences of qRT–PCR primer pairs used in the current study.

Gene	Sequence (5'–3')	Size (bp)
*β–actin*	F: AGCGAGCATCCCCCAAAGTT	285
	R: GGGCACGAAGGCTCATCATT	
*BAX*	F: TGGCAGCTGACATGTTTTCTGAC	195
	R: TCACCCAACCACCCTGGTCTT	
*BCL–2*	F: CAGGATAACGGAGGCTGGGATG	134
	R: AGAAATCAAACAGAGGCCGCA	
*Caspase-3*	F: GCGGTTGTAGAAGAGTTTCGTG	101
	R: CTCACGGCCTGGGATTTCAA	
*Caspase–8*	F: AGAGTCTGTGCCCAAATCAAC	78
	R: GCTGCTTCTCTCTTTGCTGAA	

### Immunohistochemistry

Raffin–embedded tissues were deparaffinized with xylenes and rehydrated with a gradual decrease in alcohol concentration. The slides were pretreated for antigen retrieval, conditions following the antibody (Table 2). For blocking non–specific antibodies, all specimens were blocked with 1% bovine serum albumin for 30 min at 37 °C, subsequently incubated with primary antibody at 4 °C, overnight (Table 2). The secondary antibody was following applied, using a biotinylated goat anti–mouse/anti–rabbit antibody Envision (Dako, Glostrup, Denmark) for 1 h at RT. A freshly prepared 3, 3′–diaminobenzidine (DAB) solution (Dako, Glostrup, Denmark) was added to visualize the colored reaction. Finally, the slides were immersed in distilled water, counterstained with Meyer’s hematoxylin stain, and permanently mounted. The negative control was experimental specimens, following the same procedure, except for incubating with a primary antibody. A double–blind study was performed to evaluate the immunoexpression results by certified veterinary pathologists.

**Table 2:** The immunohistochemical primary antibody, working dilutions, antigen retrieval, and positive staining pattern.

Antibody	Source	Clone/Cat. Num	Dilution	Antigen retrieval	Staining pattern
Ki–67	Leica, Newcastle, UK	MM1	1:100	Tris–EDTA (pH 9.0) Autoclave, 121 ºC, 20 min	Nucleus
PCNA	Dako, Hamburg, Germany	PC10	1:100	Citrate (pH 6.0) Microwave, 10 min	Nucleus
BAX	Sigma–Aldrich, Missouri, USA	1F5–1B7	1:100	Citrate (pH 6.0) Microwave, 10 min	Cytoplasm
BCL–2	Dako, Hamburg, Germany	124	1:100	Citrate (pH 6.0) Autoclave, 121 ºC, 20 min	Cytoplasm
Caspase-3	Abcam, Cambridge, UK	Ab4051	1:100	Citrate (pH 6.0) Autoclave, 121 ºC, 20 min	Cytoplasm, Nucleus
Caspase-8	Santa Cruz Biotechnology, Oregon, USA	8CSP03	1:100	Citrate (pH 6.0) Autoclave, 121ºC, 20 min	Cytoplasm
p53	Leica, Newcastle, UK	DO–7	1:200	Tris–EDTA (pH 9.0) Autoclave, 121 ºC, 20 min	Nucleus

In individual immunoexpression specimen, a total of ten areas were randomly captured by the digital imaging system at an HPF (40×, area equals 2.37 mm^2^). In a group of proliferative markers (Ki–67 and PCNA), p53, and Caspase-3, the number of positive cells per total nucleated cells was evaluated. The data were calculated and displayed as a percentage (%) of positive cells per total nucleated cells. On the other hand, the percentage of positive cells per 1 mm^2^ (%IHC positive area/mm^2^) within a group of related–apoptotic markers (BAX and BCL–2) were calculated by the image analysis program (NIS–Elements Analysis D, Nikon, Japan). The BAX/BCL–2 ratio was calculated by dividing the normalized ratio of BAX and BCL–2 protein expression.

### Western blot

Western blot analysis was conducted with the extracted protein from the xenograft tumors following the guideline of Minute Total Protein Extraction Kit for Animal Cultured Cells and Tissues (Invent Biotechnologies, Massachusetts, USA) and stored at − 20 °C. The lysed protein was loaded onto the 10% SDS–PAGE polyacrylamide gel electrophoresis and transferred to nitrocellulose membranes (Santa Cruz Biotechnology, Texas, USA). Thereafter, the membrane was blocked with 5% skim milk for 1 h at RT and was incubated overnight with primary antibody (Table 3) at 4 °C. Following, anti–mouse IgGk BP–HRP (Cat. Num. sc–516102, Santa Cruz Biotechnology, Oregon, USA) or anti–rabbit IgG–HRP (Cat. Num. sc–2357, Santa Cruz Biotechnology, Oregon, USA) at 1:5,000 dilution was developed as secondary antibody for 1 h at RT. For visualization, the LumiFlash Prime Chemiluminescent Substrate (Energenesis Biomedical, Taipei, Taiwan) and ChemiDoc and ChemiDoc MP Imaging Systems (Bio–Rad, California, USA) were chosen to detect and scan the results, respectively. The labeling intensity of samples was evaluated via the signal strength, using ImageJ software^64^.

**Table 3:** The primary antibodies used for WB in the study.

Antibody	Source	Clone/cat. num	Dilution
β–actin	Santa Cruz Biotechnology, Oregon, USA	C4	1:500
BAX	Sigma–Aldrich, Missouri, USA	1F5–1B7	1:500
BCL–2	Dako, Hamburg, Germany	124	1:500
Caspase-3	Abcam, Cambridge, UK	Ab4051	1:500
Caspase-8	Santa Cruz Biotechnology, Oregon, USA	8CSP03	1:500

### Statistical analysis

The one–way analysis of variance (ANOVA) and post hoc test were considered to test the difference in the quantitative variables (apoptotic index; mitotic index; Ki–67, PCNA, p53, and Caspase-3 protein expression) as well as semi–quantitative variables (BAX and BCL–2 protein expression) among groups. The qRT–PCR and western blot results were evaluated by a nonparametric test to compare the differences between the experimental groups, using the Kruskal–Wallis test. All statistical analyses were performed by the SAS software version 9 for Windows (SAS Institute, Inc., North Carolina, USA). The values were presented as mean ± standard deviation (SD). P values below 0.05 were considered the criterion for statistically significant.

### Supplementary Information

Supplementary Figure 1.

Supplementary Figure 2.

Supplementary Figure 3.

Supplementary Figure 4.

Supplementary Figure 5.

Supplementary Figure 6.

## Data Availability

The datasets used and/or analysed during the current study available from the corresponding author on reasonable request.
