# Supplementary material for: Anti-proliferative and apoptotic effect of cannabinoids on human pancreatic ductal adenocarcinoma xenograft in BALB/c nude mice model
Source: Sci Rep. 2024 Mar 18;14:6515. doi: 10.1038/s41598-024-55307-y (PMC10948389; doi:10.1038/s41598-024-55307-y)

Supplementary data Fig. 1: Gross anatomy of the xenograft tumors among the treatment groups with THC:CBD (1:6) at a dose of 1 mg/kg BW (a), 5 mg/kg BW (b), 10 mg/kg BW (c), NC group (d), and PC group (e) (Scale bar = 1 cm).

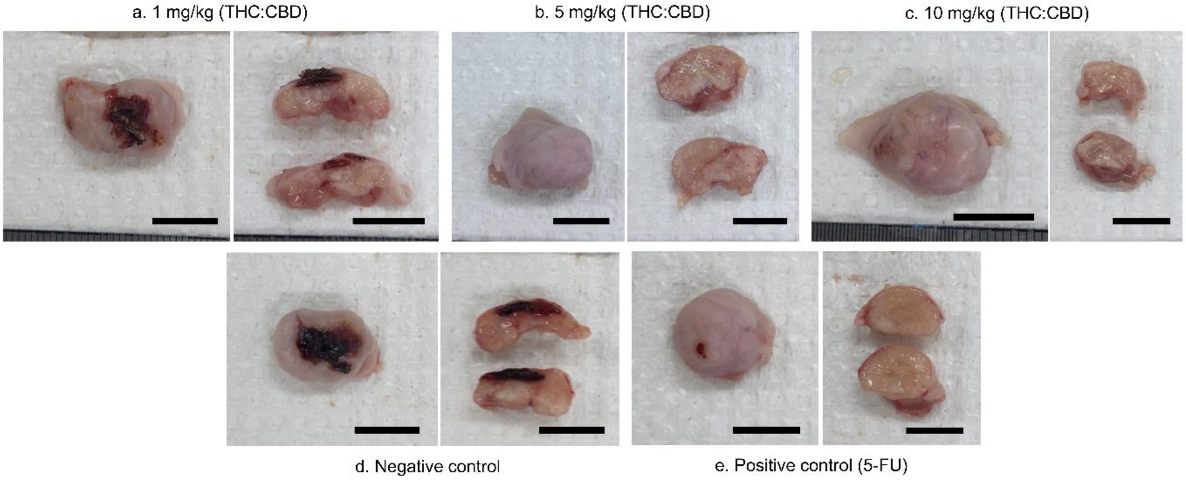

Supplement: Supplementary file 1 — Supplementary Figure 1. [file 41598_2024_55307_MOESM1_ESM.pdf]
