# Supplementary material for: Anti-proliferative and apoptotic effect of cannabinoids on human pancreatic ductal adenocarcinoma xenograft in BALB/c nude mice model
Source: Sci Rep. 2024 Mar 18;14:6515. doi: 10.1038/s41598-024-55307-y (PMC10948389; doi:10.1038/s41598-024-55307-y)

Supplementary data Fig. 2: The average tumor volume (mm^3) on day 0 (a) and day 30 (b) over the course of treatment, and percentage of tumor volume change (%) (c) among the NC group, PC group, and treatment groups (THC:CBD at a dose of 1, 5, and 10 mg/kg BW). The values were presented as mean ± SD. $^{ns}p > 0.05$ (one–way ANOVA and post hoc test).

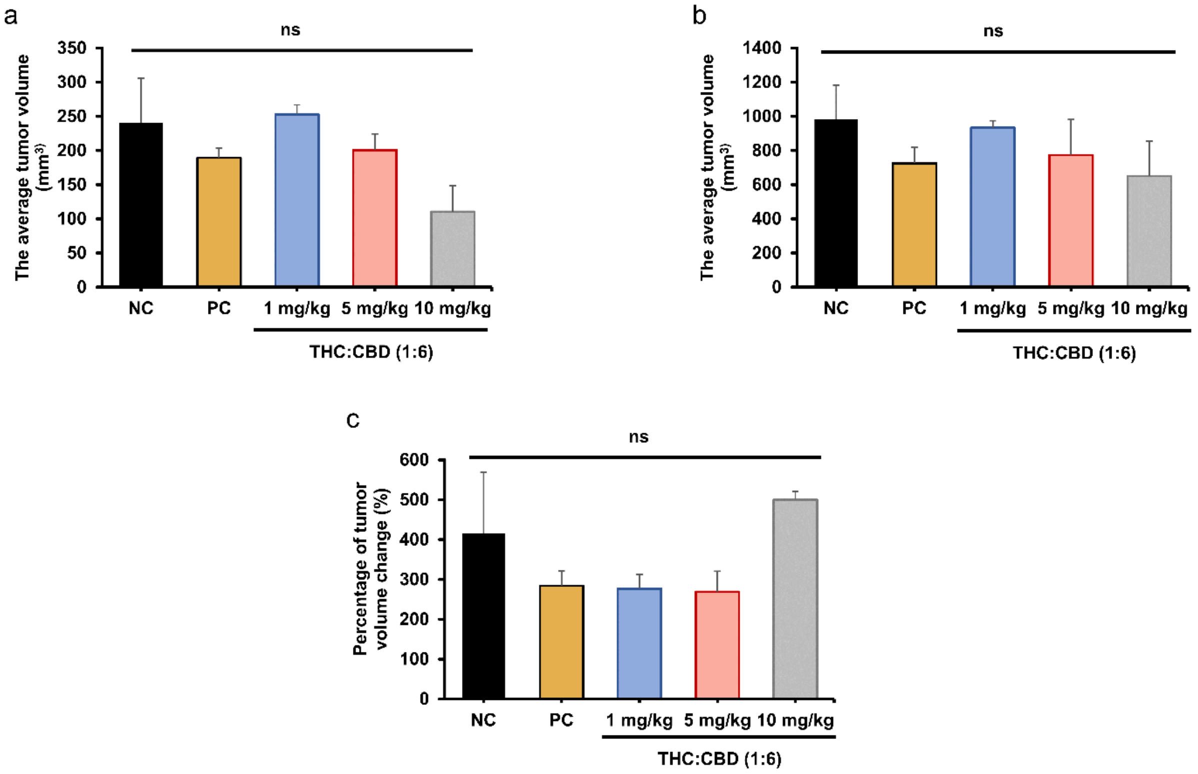

Supplement: Supplementary file 2 — Supplementary Figure 2. [file 41598_2024_55307_MOESM2_ESM.pdf]
