# Supplementary material for: Anti-proliferative and apoptotic effect of cannabinoids on human pancreatic ductal adenocarcinoma xenograft in BALB/c nude mice model
Source: Sci Rep. 2024 Mar 18;14:6515. doi: 10.1038/s41598-024-55307-y (PMC10948389; doi:10.1038/s41598-024-55307-y)

Supplementary data Fig. 4: The histological images of the lung (a), heart (b), liver (c), pancreas (d), spleen (e), and kidney (f) from the xenograft nude mice injected with Capan-2 cell line (H&E stained, 40X, scale bar = 50 μm).

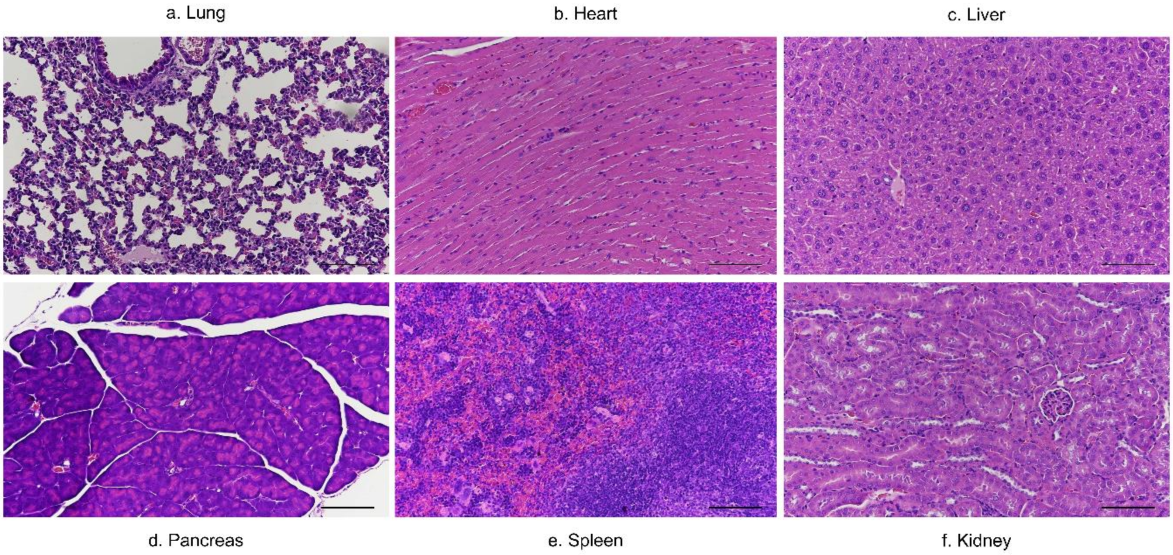

Supplement: Supplementary file 4 — Supplementary Figure 4. [file 41598_2024_55307_MOESM4_ESM.pdf]
