# Supplementary material for: Anti-proliferative and apoptotic effect of cannabinoids on human pancreatic ductal adenocarcinoma xenograft in BALB/c nude mice model
Source: Sci Rep. 2024 Mar 18;14:6515. doi: 10.1038/s41598-024-55307-y (PMC10948389; doi:10.1038/s41598-024-55307-y)

Supplementary data Fig. 5: The immunoexpression of CD31 (PECAM-1) among the NC group, PC group, and the treatment groups with THC:CBD at a dose of 1 mg/kg BW, 5 mg/kg BW, and 10 mg/kg BW. The CD31 (PECAM-1) positive labeling in the blood vessels of the border zone (a) and inside of the xenograft tumors (b) (DAB labeled, Mayer's hematoxylin counterstained, 40X, scale bar = 50 µm). The values were presented as mean ± SD. $^{ns}p > 0.05$ (one–way ANOVA and post hoc test).

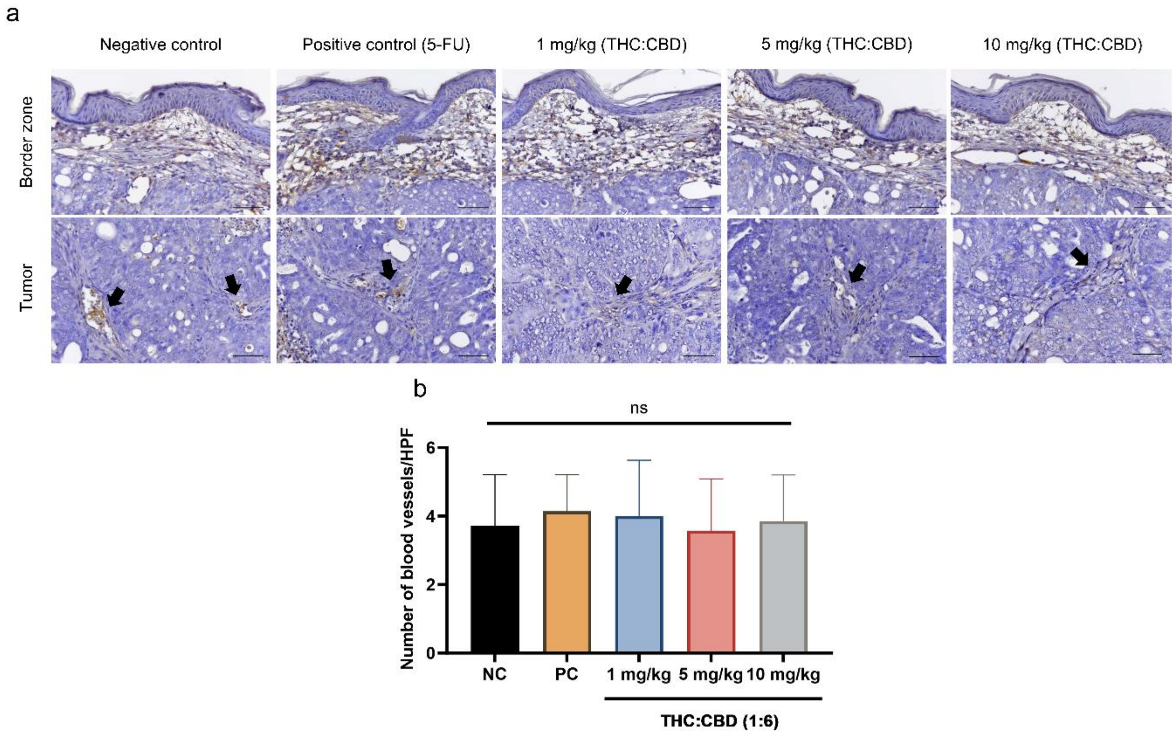

Supplement: Supplementary file 5 — Supplementary Figure 5. [file 41598_2024_55307_MOESM5_ESM.pdf]
