# Supplementary material for: Anti-proliferative and apoptotic effect of cannabinoids on human pancreatic ductal adenocarcinoma xenograft in BALB/c nude mice model
Source: Sci Rep. 2024 Mar 18;14:6515. doi: 10.1038/s41598-024-55307-y (PMC10948389; doi:10.1038/s41598-024-55307-y)
Supplement: Supplementary file 6 — Supplementary Figure 6. [file 41598_2024_55307_MOESM6_ESM.pdf]

Supplementary data Fig. 6: The electrophoretic blots of Beta actin, BAX, BCL-2, Caspase-3, and Caspase-8 antibodies. The gel system used 10% SDS-PAGE polyacrylamide gel electrophoresis and was transferred to nitrocellulose membranes (Santa Cruz Biotechnology, Texas, USA).

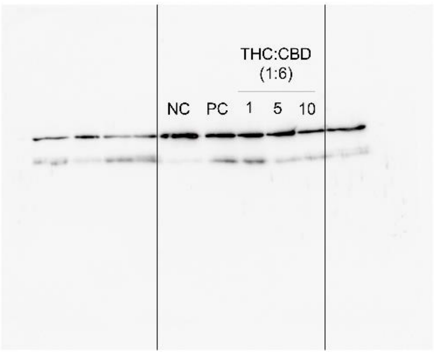

a. Beta actin

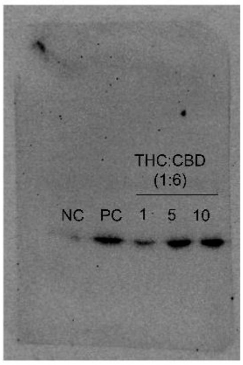

b. BAX

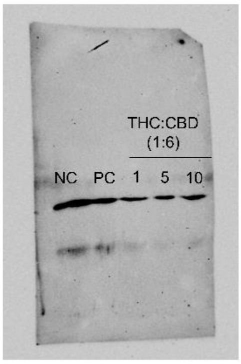

c. BCL-2

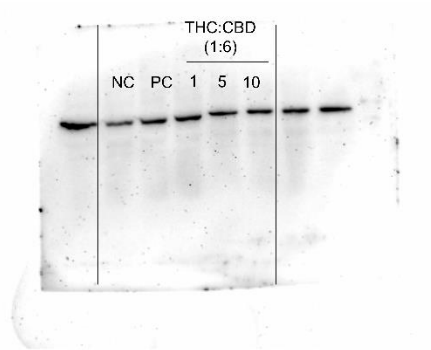

d. Caspase-3

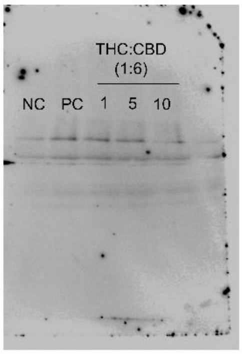

e. Caspase-8
